# Preparation of antiviral multispray with cationic antimicrobial dialkyldimethyl ammonium salt and sulfobetaine against new coronaviruses (SARS-CoV-2)

**DOI:** 10.1099/acmi.0.000631.v5

**Published:** 2024-05-31

**Authors:** Masahiko Morioka, Daisuke Shimaoka, Mamoru Ohnishi

**Affiliations:** 1 Graduate School of Science, Osaka Metropolitan University, 1-2 Garden-cho, Naka-kuSakai, Osaka 599-8570, Japan; 2 Printemps Co., Ltd, 992-1 Toichi-machi, Kashihara, Nara 634-0008, Japan; 3 2802-1, Shiroi-city, Chiba 270-1402, Hiratsuka, Maple-BioLaboratories, Japan

**Keywords:** antiviral agents, influenza virus, neutral agent, non-alcoholic agent, SARS-CoV-2

## Abstract

The novel sudden acute respiratory syndrome coronavirus 2 is an enveloped virus currently causing severe illness and death worldwide. Common antiseptics such as alcohol have some efficacy in disinfecting everyday surroundings, but development of more effective disinfectants is imperative. A series of studies focusing on cationic antimicrobials resulted in the development of a safe and effective novel coronavirus disinfectant, DEA-171, which provides ≥99.98 % inhibition of all novel coronavirus variants within 1 min.

**Impact Statement**

Vaccinations have been administered in global efforts to stem the tide of the coronavirus disease 2019 (COVID-19) pandemic, but more effective disinfectants for everyday use are needed under the assumption that we will need to coexist with the virus, as complete eradication does not seem possible. From this perspective, we focused on developing an antiviral multispray with cationic antimicrobial dialkyldimethyl ammonium salt and sulfobetaine to avoid the complications associated with skin sensitivity, instability, and environmental contamination from other types of commonly used disinfectants. Our developed antiviral multispray, DEA-171, is 99.87 % water, neutral, odourless and tasteless, and rapidly exhibits strong inhibitory effects on enveloped novel coronaviruses. This low-cost, highly safe disinfectant strongly and specifically deactivates enveloped viruses, regardless of differences in either virus gene strain or mutation, suggesting that this antiviral multispray can be easily added as part of daily efforts to neutralize the spread of COVID-19 and possibly future emerging viruses.

## Data Summary

Supplementary files regarding the single-dose toxicity testing and biological safety testing of the coronavirus disinfectant DEA-171, including replicate data for SARS-CoV-2 inhibition tests, are available via Figshare with the identifier https://doi.org/10.6084/m9.figshare.22568275.v2.[1].

## Introduction

Since its emergence, the novel sudden acute respiratory syndrome coronavirus 2 (SARS-CoV-2) has infected over 769 million people and has caused approximately 6.95 million fatalities worldwide [2]. The development of mRNA vaccines is expected to provide relief, but the efficacy of these vaccines has been limited, and complete eradication of the novel coronavirus now appears uncertain. A search for new paths toward effective coexistence may thus be necessary.

Contact infection, droplet infection and aerosol infection all play major roles in the transmission of this virus, with ethanol, hypochlorite, benzalkonium chloride, and other quaternary ammonium salts generally considered effective for preventing transmission [3]. However, ethanol requires a rather high concentration, which may incur side effects, such as facial flushing, tachycardia, and headaches, particularly for East Asians because of their susceptibility to ethanol [4]. In addition, ethanol is a skin-sensitizing substance that tends to induce skin dryness and irritation [5,7]. Hypochlorite is difficult to manage because of its instability, but toxicity is unlikely to appear. Benzalkonium chloride and cetylpyridinium chloride, if neutral, exert only weak antiviral activity and require rather high concentrations [8,10]. In addition, the use of benzalkonium chloride may cause environmental contamination, which would certainly be problematic [11]. Benzalkonium chloride and cetylpyridinium chloride have a nitrogen cation in their chemical structure, so their antiviral effects are clearly due to denaturation of the lipid membrane and cannot be expected to cause protein denaturation. Therefore, they are assumed to be highly safe. However, given the situation regarding SARS-CoV-2, the current disinfection methods are insufficient. Furthermore, these are not effective in inactivating non-enveloped viruses.

These considerations led us to focus on cationic antimicrobial didecyldimethylammonium chloride (DDAC), which is active against bacteria and viruses at lower concentrations than benzalkonium chloride and cetylpyridinium chloride. DDAC solutions at concentrations ≥0.4 wt% have been designated as deleterious in Japan because of a dermatological accident, but DDAC would pose no problem as long as the amount and duration of exposure are both within safety margins, as represented by a large difference between the levels resulting in main and side effects. Here, we report the sufficient efficacy and safety margin of DEA-171, a newly developed coronavirus disinfectant.

## Methods

The deactivation effect of DEA-171 was assessed using viruses.

### Novel coronavirus

A total of 900 µl of the DEA-171 test preparation was added to 100 µl of virus solution containing SARS-CoV-2, and then diluted with cell culture medium after 1 min to assess the immediate action and after 30 min to assess long-acting effects. Viral titres were determined by the plaque method [12] using VeroE6/TMPRSS cells. The culture lines of SARS-CoV-2 used in the present study are shown in Table 1.

**Table 1.** Culture lines of SARS-CoV-2

Type	Culture line (all owned by Yamaguchi University, Japan)
Wuhan (CHN)	NIID isolate 2019-nCoV/Japan/AI/I-004/2020
Europe	Nagasaki University isolate NG1 (unnamed)
α (UK)	QK002 hCoV-19/Japan/QK002/2020
β (ZAF)	TY8-612 hCoV-19/Japan/TY8-612/2021
γ (BRA)	TY7-501 hCoV-19/Japan/TY7-501/2021
δ (IND)	[Pango lineage B1.617.1] hCoV-19/Japan/TY11-330 P1/2021

BRA, Brazil; CHN, PR China; GBR, United Kingdom; INDIndiaIND, India;UKUnited KingdomZAF, South Africa

### Toxicity

The toxicity of DEA-171 was tested according to the standards of the Japanese Society of Industrial Technology for Antimicrobial Articles, which comply with the OECD standards TG442A [13], TG423 [14], and TG471 [15]. Specifically, TG442A, referred to as Local Lymph Node Assay: DA, is the test guideline for a skin sensitization assay; TG471 is the test guideline for a bacterial reverse mutation test; and TG423 is the test guideline for assessing acute oral toxicity via the acute toxic class method.

#### Skin sensitization tests

A biological safety (skin sensitization) study against DEA-171 was conducted according to OECD standard TG442A. Twelve mice (CBA/J) were purchased from Charles River Japan, and bred under conditions of temperature 22 °C, humidity 60 %, and artificial lighting at 12 h intervals.

##### Measurement of ATP content in lymphocytes

The test samples were applied to the ears of the mice, and after 8 days of rearing, the auricular lymph nodes were removed and the ATP concentrations were measured using a luminometer (Kikkoman C-110).

### Reverse mutation tests

The reverse mutation tests were conducted according to OECD standard TG471 using a preincubation method and the following five strains of bacteria obtained from the Biological Resource Center of the National Institute of Technology and Evaluation in Japan: TA100, TA98, TA1535, TA1537, and WP2uvrA.

#### Dose setting test

Two Petri dishes were used for each dose level (5 µg/plate). In the dose setting test, 20 µl of the suspension of each strain was inoculated into 10 ml of nutrient broth culture medium and placed in a 37 °C incubator for 12 h with shaking. Following incubation, 0.1 ml of the culture, 0.5 ml of S9Mix, and 0.1 ml of the test substance solution were mixed in a test tube and preincubated at 37 °C for 20 min. Subsequently, 2 ml of top agar with amino acids (histidine or tryptophan) was added, mixed, and spread on top of minimal glucose agar plate medium and incubated at 37 °C for 48 h. After incubation, each strain was observed under a stereomicroscope and with the naked eye for sparseness of growth, and the number of reverse mutant colonies was counted.

#### Main test

Two Petri dishes were used for each test, and these were conducted in the same manner as the dose setting test. The maximum concentration was set at 50 µg ml^−1^ (5 µg/plate), since no growth inhibition was observed in the strains in the dose setting test at that concentration.

#### Criteria for the test results

Two Petri dishes were used for each of the five strains at each dose, and data from the test substance group, the positive control, and the solvent control group were compared at five different doses.

### Mouse single-dose toxicity test

The mouse single-dose toxicity test using 12 mice was performed according to OECD standard TG423. The 12 mice were divided into the following 2 groups and subjected to the test: group I (control group, administered saline solution) and group II (administered DEA-171 100 µg/22 g mouse body weight). The mice were visually observed for signs of toxicity after administration (16 days), and after the observation period, necropsy was performed and the findings were recorded.

## Results

### Reagent adjustment

The antiviral activity of DDAC was 0.004 wt%, only 1/100th of the lower limit of deleterious concentrations (0.4 wt%), and was neutralized with aqueous NaOH solution. In the cell contact test, no dermal degradation was observed (cell survival, 104 %). Facilitation of this antiviral activity was investigated by mixing in sulfobetaine (Anhitoru 20HD; Kao, Tokyo, Japan), a low-skin-irritation amphoteric surfactant used in shampoos and other products, which is expected to heighten antiviral activities as a cocktail substance [16]. Instead of highly pure DDAC, low-cost Biosaido ST-70H (neutral; Taisho Technos, Tokyo, Japan) was used in 0.007 wt% aqueous solution (titre as DDAC, 0.004 wt% according to Taisho Technos).

When this mixture was brought to a low temperature, however, the solution clouded. To prevent such low-temperature clouding, nonionic high-molecular-weight polyether Noigen TDX80D (DKS, Kyoto, Japan) 0.02 wt% and EDTA/2Na (Chelest, Osaka, Japan) 0.002 wt% were added for cation stabilization. The chelate effect of adding Noigen TDX80D and EDTA/2Na resulted in the maintenance of antiviral activity under dilution with either soft or hard water (hardness, 70–310 mg l^−1^). The composition of the resulting final product designated DEA-171 is shown in Table 2.

**Table 2.** DEA-171 composition

Constituent	wt%
Biosaido ST-70H (Taisho Technos)	0.007
Anhitoru 20HD (Kao)	0.100
Noigen TDX80D (DKS)	0.020
EDTA/2Na (Chelest)	0.002
Water (hardness, 70–310 mg l^−1^)	99.871

### Bioactivity

DEA-171 exhibits a clear mechanism of action, is neutral, and is highly safe as a disinfectant that strongly and specifically deactivates enveloped viruses, regardless of differences in virus gene strain or mutations (Table 3). DEA-171 is also inexpensive, which could promote its widespread use.

**Table 3.** DEA-171 inhibition of SARS-CoV-2 (*n*=3)

Type	Log p.f.u. ml^−1^(0 min)	Log p.f.u. ml^−1^(1 min)	Log p.f.u. ml^−1^(30 min)	Inhibition (%)
Wuhan (CHN)	6.0801	<2.0000	<2.0000	>99.990
Europe	6.7268	<2.0000	<2.0000	>99.998
α (UK)	5.8061	<2.0000	<2.0000	>99.984
β (ZAF)	6.7310	<2.0000	<2.0000	>99.998
γ (BRA)	6.6800	<2.0000	<2.0000	>99.998
δ (IND)	6.2106	<2.0000	<2.0000	>99.994

>99.99 %1 min^3^45–81% ethanolBRA, Brazil; CHN, PR China; GBR, United Kingdom; IND, India; p.f.u., plaque-forming units; UKUnited KingdomZAF, South Africa

### Replicate data for DEA-171 inhibition of SARS-CoV-2

Replication of the SARS-CoV-2 inhibition tests (*n*=3) demonstrates the effectiveness of DEA-171. The data provided in Table 3 and the supplementary materials clearly show a decrease in the log plaque-forming units per millilitre to below the limit of detection (<2.00 log p.f.u. ml^−1^) after 1 min. This decrease is sustained at 30 min, indicating the potential long-acting effects of DEA-171.

### Biosafety

Mice exposed to DEA-171 did not exhibit any increase in body weight, erythema, or changes in ATP concentrations in their auricular lymph nodes. Based on these test results, DEA-171 was judged not to induce any skin sensitization. The reverse mutation tests using five strains of bacteria indicated that DEA-171 in both S9Mix(−) and S9Mix(+) systems did not induce any growth inhibition or reverse mutation, since the colony counts were not more than twice the counts of colonies exposed to the solvent control. Based on these results, DEA-171 was judged to be non-mutagenic. No abnormalities were observed in any of the six animals in group I or group II, indicating negative results for the mouse single-dose toxicity test.

## Discussion

The aqueous solution of DEA-171 comprises 99.87 % water, is neutral, odourless, and tasteless, and has also shown strong inhibitory effects (>99.98 % inhibition) on other enveloped novel coronaviruses (Table 3). This suggests that the cation (positive charge) of the Biosaido ST-70H deactivates enveloped viruses by attacking the anionic (negatively charged) phospholipids constituting the envelope [17]. Regarding the effects of sulfobetaine, the sulfur atom of the chemically soft base softens the nitrogen cation of the chemically hard acid of the quaternary ammonium salt, making it more compatible with the phosphoric acid of the soft base of phospholipids [18]. Moreover, in a virus such as SARS-CoV-2, in which the RNA genes are susceptible to mutation, the phospholipids of the virus envelope are derived from the infected cell [19], which enables DEA-171 to inhibit different variants of the novel coronavirus without being affected by virus mutation. DEA-171 has tested negative in all skin sensitization and mutagenicity tests and single oral administration in mice has not shown any evidence of toxicity, confirming a high level of safety. Theoretically, hand disinfection and gargle disinfection are both possible using DEA-171.

## Abbreviations

ATCC: american type culture collection
ATP: adenosine triphosphate
BRA: Brazil
CHN: pr China
DDAC: didecyldimethylammonium chloride
IND: India
SARS-CoV-2: sudden acute respiratory syndrome coronavirus 2
UK: United Kingdom
ZAF: South Africa
